# Mechanism of Ferroptosis and Its Role in Type 2 Diabetes Mellitus

**DOI:** 10.1155/2021/9999612

**Published:** 2021-06-28

**Authors:** Wenxin Sha, Fei Hu, Yang Xi, Yudong Chu, Shizhong Bu

**Affiliations:** ^1^Diabetes Research Center, School of Medicine, Ningbo University, Ningbo 315211, China; ^2^Cixi Biomedical Research Institute, Wenzhou Medical University, Cixi 315300, China; ^3^Department of Nephrology, Ningbo Medical Center Lihuili Hospital, Ningbo 315100, China

## Abstract

Ferroptosis is a novel form of nonapoptotic regulated cell death (RCD). It features iron-dependent lipid peroxide accumulation accompanied by inadequate redox enzymes, especially glutathione peroxidase 4 (GPX4). RAS-selective lethal 3 (RSL3), erastin, and ferroptosis inducing 56 (FIN56) induce ferroptosis via different manners targeting GPX4 function. Acyl-CoA synthetase long-chain family 4 (ACSL4), lysophosphatidylcholine acyltransferase 3 (LPCAT3), and lipoxygenases (LOXs) participate in the production of lipid peroxides. Heat shock protein family B member 1 (HSPB1) and nuclear receptor coactivator 4 (NCOA4) regulate iron homeostasis preventing ferroptosis caused by the high concentration of intracellular iron. Ferroptosis is ubiquitous in our body as it exists in both physiologic and pathogenic processes. It is involved in glucose-stimulated insulin secretion (GSIS) impairment and arsenic-induced pancreatic damage in the pathogenesis of diabetes. Moreover, iron and the iron-sulfur (Fe-S) cluster influence each other, causing mitochondrial iron accumulation, more reactive oxygen species (ROS) production, endoplasmic reticulum (ER) stress, failure in biosynthesis of insulin, and ferroptosis in *β*-cells. In addition, ferroptosis also engages in the pathogenesis of diabetic complications such as myocardial ischemia and diabetic cardiomyopathy (DCM). In this review, we summarize the mechanism of ferroptosis and especially its association with type 2 diabetes mellitus (T2DM).

## 1. Introduction

Ferroptosis, which was first defined in 2012, is a form of nonapoptotic regulated cell death (RCD) because it takes place without caspases, a family of cysteine proteases cleaving specific intracellular substrates leading to apoptosis [1–3]. In other words, ferroptosis occurs without the key effectors of apoptosis such as BAX, BAK, and caspases [4]. Notably, ferroptosis is dependent on intracellular iron instead of any other metals, and it is also morphologically and biochemically different from other types of RCD such as receptor-interacting protein kinase 1- (RIPK1-) dependent necroptosis (a regulated form of necrosis) and apoptosis-inducing factor 1-dependent parthanatos [5, 6]. It also does not involve the key factors of necroptosis such as MLKL, RIPK1, and RIPK3 [7].

Ferroptosis features intracellular iron overloaded and iron-dependent lipid peroxide accumulation. Additionally, ferroptosis also leads to a suppression of oxidoreductase especially glutathione peroxidase 4 (GPX4), a lipid peroxide scavenger [8]. Ferroptosis inhibitors rescue GPX4-deficient T cells from dying of lipid peroxide accumulation-induced ferroptosis [9]. Moreover, vitamin E can compensate for the lack of GPX4 as an antioxidant while vitamin C cannot probably due to its water-soluble property [9]. In some cells such as kidney cells, ferroptosis is accompanied by impaired mitochondria with reduced organelle size, disappearance of mitochondrial cristae, and rupture of the mitochondrial outer membrane [10].

Ferroptosis has been studied in many pathogenic processes since it was identified [11]. Many experiments are designed to investigate diseases related to neurons (e.g., Parkinson's disease), immune cells (e.g., diffuse B cell lymphoma), and kidney cells (e.g., acute kidney injuries) [11–14].

Ferroptosis inhibitors were found to prevent cell death better than other inhibitors such as autophagy inhibitor (3-methyladenine), necroptosis inhibitor (necrostatin-1), and apoptosis inhibitor (pan-caspase inhibitor z-VAD-fmk) [9, 15, 16]. Ferroptosis inhibitors preserved mitochondrial membrane potential (*ΔΨ*m), decreased lipid peroxidation, and reduced nonheme iron in mitochondria [15]. Furthermore, significantly lowered levels of cardiac nonheme iron and myocardial enzymes during ischemia-reperfusion injury (IRI) by ferroptosis inhibitors were observed in a DOX-induced cardiotoxicity rodent model [15]. Moreover, immune-cell infiltration into postischemic area was reduced in acute kidney injuries [17].

The role of ferroptosis in tumor development and proliferation is under intensive investigation. Some studies find that ferroptosis facilitates oncogenesis [18, 19], while other studies report inhibition of tumor cell proliferation by ferroptosis [20, 21]. An earlier study demonstrated that P53 promotes ferroptosis through inhibiting the expression of solute carrier family 7 member 11 (SLC7A11), a key component of the cystine/glutamate antiporter (*x*_*c*_^−^) system [22]. Another transcriptional target of P53, spermidine/spermine N1-acetyltransferase 1 (SAT1), was found to promote ferroptosis as well but through elevating the expression of arachidonate 15-lipoxygenase [23]. However, not all the transcriptional targets of P53 are promoters of ferroptosis. For example, the glutaminase 2 (GLS2, a transcriptional target of P53) can function as an antioxidant, which makes it potentially an inhibitor of ferroptosis [24]. Furthermore, the knockdown of GLS2 inhibits ferroptosis in fibroblasts [25]. Moreover, P53 can suppress ferroptosis through the DPP4-dependent pathway [26].

## 2. Mechanism of Ferroptosis

Intracellular iron overloaded and excessive lipid peroxides are considered the lethal elements to trigger ferroptosis [8]. The accumulation of polyunsaturated fatty acids (e.g., arachidonoyl) and reduced lipid peroxide scavenging such as the inhibition of lipid antioxidants (e.g., GPX4) lead to ferroptosis. A general review of the mechanism of ferroptosis and its association with other forms of cell death is discussed below.

### 2.1. Lipid Peroxide Production

Arachidonoyl- (AA-) OOH-phosphatidylethanolamine (PE) was identified from various phospholipids comprising the majority of lipid peroxides as the most important signal of ferroptosis [27]. Specifically, the process of AA to AA-CoA is driven by acyl-CoA synthetase long-chain family 4 (ACSL4) [28, 29], after which AA-CoA is converted to AA-PE by lysophosphatidylcholine acyltransferase 3- (LPCAT3-) promoted esterification [30]. Then, the final step to forming AA-OOH-PE requires the oxidation of AA-PE catalyzed by lipoxygenases (LOXs) [31]. The esterification and oxidation steps can also occur in a reversed order. Eventually, the uncontrolled accumulation of AA-OOH-PE induces ferroptosis. Some studies suggest that the expression of ACSL4 may, to some extent, reflect the sensitivity of a cell to ferroptosis and can be used as a marker [28].

The production of lipid peroxides is iron-dependent. Iron is involved in lipid oxidation in the following three possible ways: (I) the Fenton reaction which is an inorganic, nonenzymatic catalyzed reaction, where ferrous iron donates electrons to O_2_ or H_2_O_2_ to promote the production of reactive oxygen species (ROS) and lipid peroxides; (II) lipid autoxidation by an iron-catalyzed enzymatic reaction; and (III) AA oxidation by an iron-containing LOX-catalyzed reaction [8]. In addition, there is evidence that the cell sensitivity of ferroptosis is affected by alterations in genes regulating iron homeostasis (e.g., IREB2, FBXL5, and FTH1 influence the cell sensitivity of erastin-induced ferroptosis) and in the extracellular concentration of iron [8]. For example, in rodent models, high-iron diets increase their sensitivity to ferroptosis through increasing the extracellular concentration of iron [32, 33]. Some proteins such as heat shock protein family B member 1 (HSPB1), which can decrease the intracellular level of iron, influence the ferroptotic sensitivity as well [34]. The nuclear receptor coactivator 4- (NCOA4-) mediated ferritinophagy plays a vital role in releasing iron from ferritin. During ferritinophagy, NCOA4 binds to ferritin and delivers it to lysosomes for degradation to release iron [35]. Then, the high concentration of intracellular iron may further induce ferroptosis.

### 2.2. Lipid Peroxide Scavenging and Ferroptosis

GPX4 is the major scavenger of lipid peroxides in cells, and it is a member of the selenoprotein family. Selenium is indispensable for the oxidoreductase function of GPX4 because selenium contributes to the nucleophilicity of selenoproteins [36, 37]. Thus, a deficiency of selenium in serum or cytoplasm is likely to impair the function of GPX4, eventually causing the accumulation of lipid peroxides and then ferroptosis [38]. GPX4 has eight nucleophilic amino acids, one of which is the selenocysteine (Sec) at its active site. RAS-selective lethal 3 (RSL3) contains a chloroacetamide moiety that can react with the nucleophilic amino acid residues on GPX4, and the binding of RSL3 to GPX4 leads to inactivation of GPX4, making RSL3 a ferroptosis inducer [39].

Glutathione (GSH) is a cofactor of GPX4 and consists of three subunits: glutamate, glycine, and cysteine (a reduced form of cystine). Erastin suppresses the activity of GPX4 by inhibiting the *x*_*c*_^−^ system, which imports cystine into the cell [3]. Inadequate supply of cystine due to an inhibition of the *x*_*c*_^−^ system leads to decreased production of cysteine and the depletion of GSH, which will eventually suppress the normal activity of GPX4 in preventing ferroptosis [27].

Ferroptosis inducing 56 (FIN56) facilitates the degradation of GPX4, and 1,2-dioxolane (FINO2) inactivates GPX4 [8]. The underlying mechanisms of FIN56 and FINO2 in the degradation and inactivation of GPX4 are unknown. Additionally, FINO2 can oxidize ferrous iron to produce ROS and oxidize lipids to produce lipid peroxides, which together induce ferroptosis [40, 41].

The mevalonate pathway is of vital importance in ferroptosis. It is the most relevant cellular metabolic pathway that affects the biosynthesis of selenoproteins and other antioxidant molecules such as ubiquinol [42]. The metabolic intermediate of the mevalonate pathway, isopentenyl pyrophosphate, is indispensable for the biosynthesis of several molecules including ubiquinol [43]. Ubiquinol inhibits lipid peroxidation in the plasma membrane and blocks ferroptosis. In addition to its GPX4-degrading ability, FIN56 disturbs ubiquinone synthesis through the mevalonate pathway [44]. An antagonist of FIN56 is the ferroptosis suppressor protein 1 (FSP1, previously called AIFM2), which is one of the enzymes catalyzing the transformation of ubiquinone to ubiquinol [45]. Notably, although ubiquinone exists in almost all lipid membranes, the FSP1-dependent modification of ubiquinone can only protect against lipid peroxidation in locations exclusive of mitochondria [45].

### 2.3. Ferroptosis and Other Forms of Cell Death

#### 2.3.1. Common Features

Although it is widely acknowledged that ferroptosis is genetically, biochemically, and morphologically distinct from other RCD processes such as apoptosis, necroptosis, and parthanatos, it shares a few common features with them. As has been mentioned above, NCOA4 contributes to ferroptosis via ferritinophagy to release more iron from ferritin [35]. Additionally, there is evidence that ferroptosis is accelerated when NCOA4 expression is forced to increase by cDNA transfection [46], but limited when NCOA4 is genetically depleted [46]. However, ferritinophagy is mechanistically a selective autophagy process [47]. Thus, some studies support the idea that the activation of ferroptosis depends on the induction of autophagy [46–49]. Intrinsically disordered proteins (IDPs) and IDP regions (IDPRs) are characterized by a lack of fixed 3D structure, which allows for promiscuous interaction and regulation with other proteins via structurally unrelated messengers [50]. IDPs and IDPRs are common in apoptosis, autophagy, and necroptosis [51]. Studies have found that approximately 40% of the proteins involved in ferroptosis have some disordered regions with <30 residues and ~15% presented long-range disordered regions of >90 residues similar to IDPs in the prevalence and distribution of long disordered regions. This implied that IDPs and IDPRs are common features among ferroptosis and other RCDs [3, 51, 52]. Additionally, this result was in favor of the hypothesis that signaling proteins without a specific structure are likely to be involved in the onset of ferroptosis [52]. When SLC7A11, the key component of the *x*_*c*_^−^ system, is inhibited by erastin, the depletion of intracellular GSH [3] and the inactivation of GPX4 lead to ferroptosis [27]. However, GSH and GPX4 are both modulators in apoptosis, necroptosis, and autophagy [53, 54]. Additionally, other ferroptosis regulators such as NRF2 [55], P53 [56], and ACSL4 [28, 57, 58] may also be potential regulators of apoptosis, necroptosis, and autophagy.

#### 2.3.2. Communications through Mitochondrial Dysfunction and Endoplasmic Reticulum Stress

Mitochondrial impairment, endoplasmic reticulum (ER) stress, and inhibition of the *x*_*c*_^−^ system have been observed in ferroptosis [59–61].

Decreases in mitochondrial membrane potential (MMP) and increases in mitochondrial transition pore permeability initiate the processes of apoptosis and necrosis [62]. However, ferroptosis can also happen due to the mitochondrial dysfunction causing disturbance in mitochondrial iron homeostasis. A research shows that decreased MMP, accelerated ferritinophagy, and more ROS production in mitochondria caused by dihydroartemisinin (DHA) induce ferroptosis in acute myeloid leukemia cells [20]. However, iron-sulfur cluster assembly enzyme (ISCU), a mitochondrial protein, can alleviate the toxicity of DHA by regulating iron metabolism, preserving mitochondrial function, and increasing the level of GSH [20]. Thus, it is possible that when mitochondria are damaged, ferroptosis occurs due to the dysregulation of mitochondrial iron homeostasis along with other RCD processes.

Oxytosis, also named oxidative glutamate toxicity, is a glutamate-induced cell death mediated by a block of the *x*_*c*_^−^ system [63]. Notably, dysfunctional *x*_*c*_^−^ system causing GSH depletion also happens in ferroptosis [8, 20, 64]. Research demonstrated that ferroptosis shares part of its pathway with oxytosis where BID (a proapoptotic protein) mediates cell death from mitochondrial dysfunction [10, 65]. Specifically, in both cases of BID knockout and using BI-6c9 (a BID inhibitor), the erastin-induced (ferroptosis) and the glutamate-induced (oxytosis) cell deaths were blocked, whereas overexpressed BID promoted cell death [65]. The ferroptosis inhibitor ferrostatin-1 (Fer-1) partially prevents oxytosis through blocking BID translocation to mitochondria but it is unable to rescue damages caused by BID that are already in mitochondria. However, BI-6c9 can counteract BID before and after its translocation to mitochondria. The BID pathway in ferroptosis and oxytosis is also detected in mouse embryonic fibroblasts [65].

Although there is not enough evidence to suggest that ferroptosis further induces apoptosis in cells, there are experiments demonstrating that ferroptotic agents not only induce ferroptosis but also enhance tumor necrosis factor-related apoptosis-inducing ligand- (TRAIL-) induced apoptosis [56]. TRAIL is capable of inducing apoptosis in malignant human cells but not in the majority of normal cells, which is a pharmacologically preferred property [66]. When treating human cancer cells with artesunate (ART, a ferroptosis inducer) or erastin together with TRAIL, the cytotoxicity of TRAIL is enhanced through an increase in ER stress-induced p53-independent PUMA (p53 upregulated modulator of apoptosis) expression [56]. However, TRAIL has no effects on ferroptotic agents-induced lipid peroxidation when they are supplied to cancer cells simultaneously. Additionally, ferroptosis inhibitors Fer-1 and liproxstatin-1 cannot prevent ER stress or the synergistic cytotoxicity of ER stress and TRAIL [56]. Interestingly, iron chelator deferoxamine (DFO) is the only ferroptotic inhibitor that can suppress ART-induced (but not erastin-induced) ER stress [56]. The varied results of different ferroptosis inhibitors indicate two things: one is that although ferroptosis and apoptosis may share common pathways leading to cell death, they are independent; the other is that the different ferroptosis inhibitors may act in different ways. Thus, drug combination of inhibitors of ferroptosis and apoptosis is suggested in preventing cell death to achieve better protective effect.

## 3. Ferroptosis in Diabetes Mellitus

It was reported that 9.3% of Americans (approximately 29.1 million persons) were diagnosed with diabetes in 2014 [67], and it was estimated that 86.1 million adults in the United States have prediabetes [67]. Islet *β*-cell failure and peripheral insulin resistance are the main pathological manifestations of diabetes mellitus. To be specific, type 1 diabetes mellitus is attributed to an absolute insulin deficiency due to *β*-cell destruction, while type 2 diabetes mellitus (T2DM) is attributed to a progressive insulin secretory defect on the background of insulin resistance [68]. The complications of T2DM are implicated in almost every tissue of the body especially the cardiovascular system, the optical system, and the renal system [69]. The pathogenesis of T2DM is still largely unknown, and so is its association with ferroptosis. To date, only a small number of studies have explored the relationship between ferroptosis and T2DM. In this section, we will review the potential role of ferroptosis in T2DM.

Pancreatic islet *β*-cells are susceptible to ferroptosis. Some studies indicate that pancreatic *β*-cells express a low level of antioxidant enzymes such as superoxide dismutase (SOD), GSH peroxidase, and catalase [70]. Hence, they are susceptible to oxidative stress because ROS is prone to accumulate. ROS accumulation can trigger many forms of deterioration, including ferroptosis. A study showed significantly reduced glucose-stimulated insulin secretion (GSIS) capacity in human islet *β*-cells when they were treated with the ferroptosis inducer erastin (but not RSL3) in vitro [71]. Conversely, pretreatment with a ferroptosis inhibitor, Fer-1 or DFO, rescued the damage to GSIS [71].

ROS concentration in *β*-cells can be raised by arsenic. There is evidence that chronic exposure to arsenic is a significant risk factor for developing T2DM [72, 73]. An experiment using MIN6 cells, mice pancreatic islet *β*-cell line, showed that ferroptosis was involved in pancreatic islet *β*-cells injury caused by arsenic via increased intracellular iron concentration and accumulated lipid peroxides resulting in RCD. Specifically, mitochondrial damage caused by NaAsO2 produced excessive mitochondrial ROS (MtROS), which further led to MtROS-dependent autophagy and increased intracellular concentration of iron. This eventually resulted in ferroptosis in MIN6 cells and impaired insulin secretion [74]. In addition, blocking this MtROS-mediated pathway promoted the insulin secretion of islet *β*-cells [74]. Another study showed that curcumin and (-)-epigallocatechin-3-gallate (EGCG), two polyphenols, can protect murine MIN6 pancreatic *β*-cells from iron toxicity and erastin-induced ferroptosis by acting as iron chelators and preventing GSH depletion and lipid peroxidation [75]. In addition to their iron-chelating properties, some polyphenols may also function as an antioxidant [76].

Taken together, ferroptosis is associated with insulin secretion dysfunction in pancreatic *β*-cells. The function of pancreatic islets can be impaired by proferroptotic factors even before *β*-cells die. Thus, monitoring and control of ferroptosis-related factors may facilitate early diagnosis and therapy of T2DM.

### 3.1. Induction of Iron Accumulation in T2DM

Ferroptosis is directly associated with the body level of ferritin. Epidemiological studies have revealed the potential association between excessive body iron storage and T2DM [75, 77, 78]. The development of insulin resistance in the association between iron and T2DM has been revealed in early studies [78, 79]. The current hypothesis is that the higher the body iron storage, the higher the risk in developing T2DM [80, 81]. For example, a mouse model of hereditary hemochromatosis which resulted in iron overloaded revealed iron deposition, increased fatty acid oxidation, and decreased glucose oxidation in the skeletal muscle, which eventually aggravates insulin resistance [78, 79]. So hemochromatosis is possibly related to diabetes. Nonetheless, a longitudinal study on overweight/obese individuals with an impaired glucose tolerance test showed no association between body iron storage and the incidence of diabetes [82, 83]. A possible explanation is that in this case, obesity is the dominant factor in diabetes development.

From the perspective of ameliorating diabetes, there are studies demonstrating that improved insulin secretion and insulin sensitivity as well as better control of blood glucose were observed after reducing the level of body iron storage [84, 85].

However, it cannot be ignored that calculating the body iron storage from the level of serum ferritin is not fully reliable, because ferritin also rises in inflammation, cancer, and liver disease [86]. Thus, it is still unclear whether high ferritin in the blood is a cause or result of diabetes [78]. Nevertheless, serum ferritin level test can still be used in the early diagnosis of T2DM and gestational diabetes [87–90].

**Figure 1:**
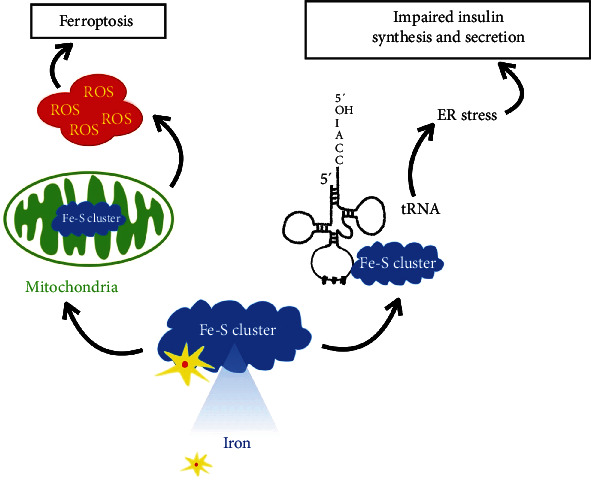
The pathway of Fe-S cluster deficiency in T2DM. Fe-S cluster deficiency affects the modification and function of tRNA, resulting in mistranslated proinsulin. This mistranslation can lead to ER stress and impaired insulin synthesis and secretion. Also, iron accumulation and more ROS production due to Fe-S cluster deficiency in mitochondria promote lipid peroxidation, further inducing ferroptosis in *β*-cells. Additionally, ROS directly impairs insulin synthesis and secretion.

Iron-sulfur (Fe-S) clusters are contained in the enzymes involved in the modification of tRNA at position 37 [91]. Since most tRNAs need to be modified at position 37, which is adjacent to the 3′ position of the anticodon, Fe-S clusters are indispensable for tRNA modification and protein translation. For example, Fe-S clusters are involved in forming 2-methylthio-N6-threonyl carbamooyladenosine (ms^2^t^6^A) at position 37 in tRNA^Lys^(UUU) [59, 92]. When cells lack ms^2^t^6^A37 due to Fe-S cluster deficiency, proteins including proinsulin are mistranslated, which may further trigger ER stress [59, 93]. The abnormal insulin synthesis and secretion as well as ER stress due to the mistranslated proinsulin contributes to the development of T2DM [59]. Meanwhile, Fe-S clusters regulate mitochondrial iron homeostasis in mitochondria [91]. The mitochondrial iron accumulation caused by Fe-S cluster deficiency can further lead to iron-mediated ROS generation inside mitochondria [94] and then ferroptosis cell death due to lipid peroxides accumulation [20]. This induction of ferroptosis causes RCD in *β*-cells accelerating the development of T2DM [74]. Also, ROS directly impairs insulin synthesis and secretion in the development of T2DM [95] (Figure 1).

### 3.2. Double-Faceted Effects of Selenoproteins and ACSL4 on T2DM Promotion

Selenoproteins and ACSL4 are groups of enzymes involved in the regulation of lipid peroxides. These enzymes are not limited in ferroptosis. Actually, they are active in many physiological reactions and potentially have an effect on the pathogenesis of T2DM.

Although ROS is often considered a contributor to the pathogenesis of T2DM, ROS is intrinsically a signaling molecule that is indispensable in the downstream insulin-induced signaling pathway to assist glucose disposition. The Gpx1^−/−^ mice were generated through inserting a 1.1 kb fragment of neomycin resistance gene cassette into the unique SacII site within the first exon of Gpx1. This Gpx1^−/−^ mice model plays a unique role in elucidating the contribution of Gpx1 in the protection against situations of oxidative stress [96, 97]. An experiment on Gpx1^−/−^ mice demonstrated the positive effect of ROS in enhancing insulin sensitivity [98]. GPX1 and GPX4 are both from the same selenoprotein family and are both antioxidant enzymes. This explains why GPX1 knockout resulted in elevated ROS production, but how does ROS promote insulin sensitivity? Further research showed that ROS activated the PI3K/Akt signaling pathway, which is key in the downstream of the insulin-induced pathway leading to glucose disposition [98, 99]. This was especially observed in the skeletal muscle. Thus, GPX1 knockout increased insulin sensitivity through ROS-mediated enhancement of the PI3K/Akt signaling pathway, leading to more glucose uptake in muscle [98]. Although GPX1 and GPX4 are from the same protein family, the deficiency of GPX4 in islet *β*-cells may trigger T2DM due to ferroptosis while the deficiency of GPX1 enhances peripheral insulin sensitivity.

**Figure 2:**
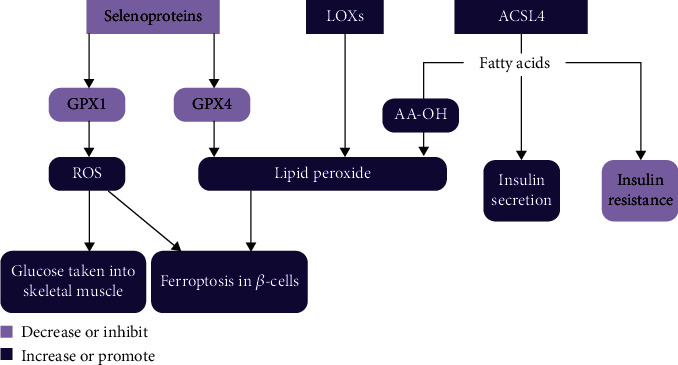
Selenoproteins, LOXs, and ACSL4 affect pathogenesis of DM in different ways. GPX1 and GPX4 are both selenoproteins. A decrease in GPX1 or GPX4 causes accumulation of ROS and lipid peroxides in *β*-cells. Excessive ROS and lipid peroxides in turn induce ferroptosis in *β*-cells. ROS also promotes glucose intake into skeletal muscles. An increase in LOXs or ACSL4 produces excessive lipid peroxides and then ferroptosis in *β*-cells as well. Although ACSL4 can promote insulin secretion in *β*-cells, it aggravates peripheral insulin resistance.

As has been mentioned above, ACSL4 catalyzes AA into AA-CoA to produce lipid peroxides in ferroptosis. Upregulated expression of ACSL4 was observed in mice fed with a high-fat diet [100]. In that same study, adipocyte-specific ablation of ACSL4 (Ad-KO) in mice fed with a high-fat diet was found to protect the mice from developing insulin resistance [100]. However, another experiment demonstrated that ACSL4 proteins are present in *β*-cells in human and rat pancreatic islets and are more concentrated around insulin secretory granules and mitochondria than at other intracellular organelles [101]. This result suggests that ACSL4 participates in insulin secretion by modifying fatty acids in insulin secretory granules and mitochondria [101]. Thus, more experiments on the function of ACSL4 are needed to delineate the role of ACSL4 in T2DM pathogenesis and the potential of targeting ACSL4 in the treatment of T2DM (Figure 2).

### 3.3. Ferroptosis and Myocardial Diseases in T2DM

Under diabetic conditions, dysregulation blood sugar levels may cause complications in almost every tissue of the body especially the cardiovascular system, the optical system, and the renal system [69]. In fact, evidence indicates that ferroptosis is involved in IRI [15, 102, 103]. Hence, organs that are more likely to suffer from ischemia (e.g., the cardiomyocytes) in T2DM are more likely to undergo ferroptosis [102, 104].

The incidence of myocardial ischemia in diabetes is 2.45 to 2.99 times higher than that in nondiabetes [105]. The prevalence of silent myocardial ischemia is 20~30% in asymptomatic patients with T2DM [106]. Thus, T2DM can be a risk factor for myocardial IRI. It has been confirmed that iron overloaded happens in cardiomyocytes and nonmyocytes during IRI in vivo [107]. This study also confirmed that ferroptosis can be induced in primary cultures of adult mouse cardiomyocytes using either Fe^3+^ or either of two ferroptosis inducers: erastin or RSL3 [107]. A recent study has demonstrated that inhibition of ferroptosis by Fer-1 during myocardial ischemia and reperfusion in diabetic rats can alleviate ER stress and myocardial damage with further in-depth experiments indicating that ferroptosis is involved in myocardial IRI through ER stress [102]. These findings suggested that during the process of myocardial IRI, ferroptosis and ER stress enhanced each other in causing myocardial injury [102]. Another study also demonstrated that Fer-1-treated mice showed reduced infarct size and serum markers of myocardial injury in IRI in vivo [15]. Some other ferroptosis inhibitors also have the ability to alleviate IRI. For example, iron chelator DFO reduced infarct size [15, 25], and the overexpression of GPX4 in mitochondria preserved mitochondrial and contractile function after global ischemia/reperfusion (I/R) in isolated, perfused mouse hearts [108]. However, these studies only show that ferroptosis is involved in IRI, and the specific role of ferroptosis needs more exploration.

Diabetic cardiomyopathy (DCM) is not an uncommon disease among diabetes [109]. Oxidative stress and impaired antioxidant system under the hyperglycemic condition are the basis of the pathogenesis of DCM [55, 110, 111]. Since an imbalance of the antioxidant system usually leads to excessive ROS production, consequent ferroptosis, apoptosis, inflammation, and fibrosis may occur in myocardial cells [55]. Nuclear factor-erythroid 2 p45-related factor 2 (NRF2) plays a pivotal role in maintaining cellular redox by its regulation of multiple antioxidants [112]. The regulations include almost all the genes encoding antioxidants implicated in ferroptosis such as the genes for glutathione regulation, NADPH regeneration, lipid peroxidation, and iron regulation [113, 114]. Loss of selenoprotein expression is in part counteracted by the NRF2-dependent system [38]. Recent studies indicate that activating NRF2 to suppress ferroptosis can be a potential therapeutic target for DCM in animal models [115, 116]. Rutin is both an NRF2 activator and a phytochemical with multiple pharmacological activities including antidiabetic, antioxidative, and free radical-scavenging bioactivities. It has been shown to be effective in alleviating DCM in animal models of both type 1 and type 2 diabetes [117–119]. However, how NRF2 activation alters ferroptosis in the pathogenesis and development of DCM remains unclear [119].

## 4. Conclusion

Ferroptosis features intracellular iron overloaded and lipid peroxides. Iron and enzymes such as LOXs, ACSL4, and LPCAT3 together produce excessive lipid peroxides (especially AA-OOH-PE) in cells, causing ferroptosis. Thus, the levels of iron and those lipid oxidases may implicate the cellular sensitivity to ferroptosis. GPX4 functions as a lipid reductase. Molecules inhibiting the activation of GPX4, such as RSL3 via combining with its active site, erastin via depleting its cofactor GSH, and FIN56 via facilitating its degradation, also induce ferroptosis. Moreover, IDPs/IDPRs, mitochondrial dysfunction, and ER stress are what correlate ferroptosis with other RCDs.

Ferroptosis is widely investigated in its role in both physiologic and pathogenic processes but its role in T2DM has not been extensively studied. Since *β*-cells lack a strong antioxidation mechanism, they are possibly susceptible to ferroptosis. Evidence has shown the involvement of ferroptosis in GSIS impairment and arsenic-induced pancreatic islet cell damage. High concentrations of serum iron can be a risk factor of developing T2DM. Hence, monitoring and control of factors related to ferroptosis might be promising measures in the early diagnosis and therapy of T2DM. However, additional studies on ferroptosis and its involvement in T2DM are needed to identify the appropriate diagnostic and therapeutic targets.

## Data Availability

All data used and/or analysed during the present study are available from the corresponding author on reasonable request.
